# Urban Climate Policy and Action through a Health Lens—An Untapped Opportunity

**DOI:** 10.3390/ijerph182312516

**Published:** 2021-11-27

**Authors:** Audrey de Nazelle, Charlotte J. Roscoe, Aina Roca-Barcelό, Giselle Sebag, Gudrun Weinmayr, Carlos Dora, Kristie L. Ebi, Mark J. Nieuwenhuijsen, Maya Negev

**Affiliations:** 1 Centre for Environmental Policy, Imperial College London, London SW7 1NE, UK; 2 MRC Centre for Environment and Health, Department of Epidemiology and Biostatistics, School of Public Health, Imperial College London, Norfolk Place, London W2 1PG, UK; a.roca-barcelo@imperial.ac.uk; 3 Landmark Centre, Harvard T.H. Chan School of Public Health, Harvard University, Boston, MA 02215, USA; croscoe@hsph.harvard.edu; 4 International Society for Urban Health, New York, NY 10003, USA; gsebag@isuh.org (G.S.); cdora@isuh.org (C.D.); 5 Institute of Epidemiology and Medical Biometry, Ulm University, 89081 Ulm, Germany; Gudrun.Weinmayr@uni-ulm.de; 6 Center for Health and the Global Environment (CHanGE), University of Washington, Seattle, WA 98195, USA; krisebi@uw.edu; 7 Institute for Global Health (ISGlobal), 08003 Barcelona, Spain; mark.nieuwenhuijsen@isglobal.org; 8 CIBER Epidemiología y Salud Pública (CIBERESP), 28029 Madrid, Spain; 9 Department of Experimental and Health Sciences, Universitat Pompeu Fabra (UPF), 08003 Barcelona, Spain; 10 School of Public Health, University of Haifa, Haifa 31905, Israel; mnegev@univ.haifa.ac.il

**Keywords:** climate change, co-benefits, systems thinking, co-production, cities

## Abstract

Motivated by a growing recognition of the climate emergency, reflected in the 26th Conference of the Parties (COP26), we outline untapped opportunities to improve health through ambitious climate actions in cities. Health is a primary reason for climate action yet is rarely integrated in urban climate plans as a policy goal. This is a missed opportunity to create sustainable alliances across sectors and groups, to engage a broad set of stakeholders, and to develop structural health promotion. In this statement, we first briefly review the literature on health co-benefits of urban climate change strategies and make the case for health-promoting climate action; we then describe barriers to integrating health in climate action. We found that the evidence-base is often insufficiently policy-relevant to be impactful. Research rarely integrates the complexity of real-world systems, including multiple and dynamic impacts of strategies, and consideration of how decision-making processes contend with competing interests and short-term electoral cycles. Due to siloed-thinking and restrictive funding opportunities, research often falls short of the type of evidence that would be most useful for decision-making, and research outputs can be cryptic to decision makers. As a way forward, we urge researchers and stakeholders to engage in co-production and systems thinking approaches. Partnering across sectors and disciplines is urgently needed so pathways to climate change mitigation and adaptation fully embrace their health-promoting potential and engage society towards the huge transformations needed. This commentary is endorsed by the International Society for Environmental Epidemiology (ISEE) and the International Society for Urban Health (ISUH) and accompanies a sister statement oriented towards stakeholders (published on the societies’ websites).

## 1. Climate and Health—Missed Opportunities for Synergies

The frequency, intensity and severity of extreme weather events are rapidly increasing and are projected to continue rising unless humanity takes strong and immediate action. The public health impacts of climate change are widely acknowledged in research [1]. In this summer alone, communities across the world have experienced first-hand the devastating consequences of climate change: unprecedented record high temperatures in Canada, devastating flooding in China and Germany, droughts in Southern Africa and heat waves and accompanying wildfires in Canada, the United States, Russia, Greece, and Turkey. These examples are not anomalies, but our new routine reality. Thousands of people have been displaced, bereft of their possessions, injured, fallen ill, or even killed. The link between climate change and health seems obvious, with a rapidly rising death toll, serious physical and mental health impacts, and perilous social and economic consequences. Health, however, is rarely considered a direct driver for climate mitigation and adaptation policies. Structural health promotion through “higher order interventions” that address institutional, community, and policy levels, can have direct impacts on exposure levels to environmental hazards and also act as a catalyst for social and behavioral changes [2]. In this piece, we argue that the consideration of health, particularly of structural approaches to health promotion, is an untapped opportunity in climate action with the potential to significantly improve health outcomes.

The co-benefits of climate action are indisputable. A recent systematic review has identified over twenty health modeling studies that have quantified multiple health co-benefits from climate mitigation strategies targeting land use, transport, buildings, and waste management [3]. Such health-promoting climate change strategies are effective in addressing climate change while simultaneously tackling some of the greatest public health challenges of our century, such as the obesity and diabetes epidemics. For example, urban planning and design solutions that reduce car use and provide opportunities to walk, bike, and take public transportation, allow individuals to lead active lifestyles while also limiting air pollution, reducing asphalt surface, improving access to greenspaces, increasing social interaction and reducing greenhouse gas emissions. Health impact models have estimated that mode shifts such as those which encourage physical activity would reduce mortality rates by 4 to 45 times compared to interventions such as electric cars that reduce air pollution alone. This makes strategies that integrate health co-benefits far more impactful for cities and societies than purely technological solutions [4,5,6,7]. Few studies have quantified co-benefits beyond air quality, greenhouse gas (GHG) emissions, and physical activity. Barcelona’s superblock model, however, provides a good example of how reducing motorized transport can promote sustainable mobility and an active lifestyle, augment urban greening, and mitigate the effects of climate change [8]. Through these different pathways to health (reduced heat, noise and air pollution exposure, and increased physical activity and greenspace exposure), the superblock model is estimated to result in an average increase in life expectancy for the Barcelona adult population of almost 200 days, and boost the city’s annual economic revenue by 1.7 billion euros [8]. Improvements in other sectors, such as waste, construction, and energy, also offer excellent opportunities for both health and the environment. In Accra, Ghana, for example, where waste contributes to 14% of GHG emissions, halving GHG emissions through waste management strategies could save 120 lives in 2030 from air pollution reduction [9]. Similarly, improved waste management scenarios in Nairobi were estimated to save 4.35 million US dollars from air quality improvements [10]. In Suzhou, China, a combination of strategies in transport fuel economy and industrial and buildings energy could lead to 50% reduction in the burden of diseases associated with air quality improvements, accompanied by a commensurate reduction in GHG emissions [11]. Most climate policy health co-benefit assessments have focused on quantifying air quality-related impacts [3]. The 2021 World Health Organization air quality guidelines emphasize the crossroads between air quality and climate action and urges combined approaches to protect health [12]. There are, however, multiple further co-benefits that may emerge from these strategies that have not necessarily been quantified. For example, soil protection and biodiversity can result from improved waste management [9] and reducing car use through urban design can potentially lead to the creation of new public, open and natural spaces with associated improvements in biodiversity, flood, and heat management, and opportunities for socializing and addressing loneliness. These, in turn, lead to mental and physical health benefits [13,14,15].

Cities are unique settings to tap into the health co-benefits of climate action [16]. Firstly, cities are expected to suffer immensely from the impacts of climate change, and therefore have much to gain through mitigation efforts [17]. Secondly, cities are home to most of the world’s population and therefore even small changes in their structure and functioning can result in widespread and substantial health effects, such as through small changes in air pollution [18]. Moreover, cities have unique characteristics that make them more vulnerable to certain disasters such as heatwaves, due to the urban heat island effect, or flooding, due to impervious surfaces. Finally, cities are the largest contributors to GHG [17]. This makes them a target to fast-track the reduction in carbon emissions. Despite some cities leading the change [19], many do not prioritize tackling climate change due to the lack of perceived direct and immediate local benefits of climate policies [20]. In short, urban climate change mitigation and adaptation strategies have the potential to prevent worsening global warming, and by doing so, to promote population health. We focus this commentary on urban climate change strategies, noting, however, that the urban–rural dichotomy is to a certain extent artificial. Beyond urban centers, policies around transport, nutrition, and fossil fuel and energy, for instance, are equally relevant for the maximization of co-benefits across all population densities.

Health promotion shows good alignment across climate change mitigation and adaptation strategies, yet this synergy is often disregarded. Urban policies tend to be developed using piecemeal and siloed approaches. For example, environmental departments may have air quality targets, health departments and medical institutions may have exercise and healthy nutrition targets and, in cities with a climate agenda, there may be a target for GHG reduction. However, cities rarely combine their ambitions to achieve these interrelated targets most effectively, missing an opportunity for win–win results. Consequently, synergistic solutions to tackle the top public health risk factors, preserve the environment and tackle climate change are often not implemented. In this context, urban planning is increasingly recognized as having a determining role in many health, ecology, and climate endpoints. Unfortunately, planners are not often equipped with the relevant knowledge or tasked with achieving health promotion, nature conservation, or climate mitigation or adaptation targets and opportunities are missed.

The absence of health in the climate change action paradigm finds its roots in the way we view and frame climate action through international agreements. The challenge of incorporating health into climate negotiations is partially because climate change started as, and primarily remains, an environmental issue [21]. This became clear at the 26th Conference of the Parties (COP26) held this November in Glasgow, United Kingdom. COP26 followed a special report from the WHO, which emphasized the need to put health at the center of the climate agenda [22]. COP26 attendees were tasked with building climate-resilient health systems [23], several side events were hosted at the Blue Zone Health Pavilion, and a parallel conference was hosted by the WHO. Despite these efforts, however, health discussions remained peripheral and, once again, were not on the negotiation tables. COP26 agreements did not include health targets, nor consider health co-benefits in the decision-making process. 

In this context, more than ever, researchers have a responsibility to engage in society and deliver evidence that meets the needs of our complex planet and urban systems, which includes assessing means to maximize health co-benefits of future climate policies. Structural health promotion, as a direct benefit or a co-benefit of climate change strategies, is an untapped opportunity to engage members of the public, stakeholders, and policy makers in ambitious climate actions that meet the needs of local communities. This commentary highlights and explores barriers for the integration of health in urban climate change policymaking and actions that were recently explored in a systematic review [3] and suggests a way forward to promote healthy city planning as a major climate mitigation and adaptation strategy. This research-focused commentary is endorsed by the International Society for Environmental Epidemiology (ISEE) and the International Society for Urban Health (ISUH), and accompanies a sister statement aimed at stakeholders, published on the societies’ websites [24].

## 2. Climate Change and Health Co-Benefits Research: Challenges and Opportunities

In recent years, there have been efforts to develop new tools for assessing the health impact of climate and other policies which have broadened our understanding of climate-health co-benefits. Health impact assessments (HIAs) are a practical approach used to judge the potential health effects of a policy, programme or project on a population, particularly on vulnerable or disadvantaged groups, Along with related models such as comparative risk assessments or integrated HIAs [25], these tools have demonstrated the potential health co-benefits of urban climate strategies that were highlighted in the previous section [3]. Nonetheless, this policy-oriented research has not been followed by widespread policy action [26,27,28]. The lack of uptake of research on urban climate change policies can be attributed to a complex myriad of factors. From a research perspective, there are two major aspects to consider:

(1) Are researchers producing evidence sufficiently relevant to local policymakers, decision makers and stakeholders?
(2) Are local leaders, mayors, and their staff able to access high-quality research that is translated in a way that communicates to them and to their constituents effectively?

### 2.1. Producing Relevant Evidence

Policymaking in local government is a complicated process, a complexity which is poorly acknowledged in research on urban climate change policies. Creating new programs, plans and initiatives is multidimensional and involves competing interests, social dynamics, and the interconnected pathways between urban planning, social services, sustainability, education, transportation, parks and recreation, economic development, and health, among others. Decision makers must balance various goals, lobbies, time constraints related to election cycles, news events, human emotions, budgets, etc. One obvious source of complexity is the sheer breadth of environmental, social and health implications of an urban planning program, initiative, plan, or strategy. 

Government and local government departments and administrative institutions are often disconnected and siloed, resulting in competing agendas and budgets rather than shared goals and an efficient deployment of funds. This lack of interconnectedness discourages agency heads from investing in initiatives and policies that do not directly impact their individual targets, often inadvertently condemning any strategy or plan with co-benefits across multiple agencies to failure. Research often mirrors the siloed thinking of policy and practice. The piecemeal approach used by researchers is rooted in the traditional academic structure of disciplines and remains inflexible, being further encouraged by the characteristics of the funding landscape [29]. Few existing research evaluations or assessments of policies or actions quantify more than one or two impacts at a time [3]. While several authors have developed comprehensive conceptual frameworks to encourage holistic and dynamic assessments of climate change policies [30,31], these have remained conceptual and have not been applied in real world settings to provide useful concrete quantitative or mixed-methods assessments of mutual co-benefits that would be useful for decision makers [32]. 

A lack of political will and leadership poses a great threat to the implementation of ambitious integrated climate change and health policies [28,33]. Policymakers must contend with vested interests, powerful lobbies, and a common misperception that public health policies restrain economic growth [27,34,35]. Transformative action towards integrated solutions to health will thus rely on understanding the powers at play and the imperatives of electoral cycles, identifying leverage points, and developing convincing arguments tailored to these realities. Public health research has often been criticized in the past for its lack of engagement in politics [28,36], limiting the usefulness and uptake of its outputs. To be policy-relevant, research must integrate the complex dynamics of decision-making processes [37].

The integration of social complexity is lacking in research on urban climate change and health. Social scientists, policy analysts, and urban planners, are rarely involved in public health research, although much of this research is conducted predominantly to improve the evidence base for policymaking. Understanding how people interact with their social, political, cultural, and physical environment is essential to predict shifts in behaviors and world views, which will ultimately shape the way cities and individuals operate [38]. Planetary health concepts and the social-ecological model of health are sometimes referenced in conceptualizations of the interplay between individual, social, environmental, and physical factors affecting health [39]. In practice, however, socio-ecological constructs have not been applied in evaluations of real-world plans [26,39]. Studies with narrow lenses that do not consider the complex dynamics and interrelationships present in cities make extrapolations to real life settings difficult and policy-relevance limited.

### 2.2. Research Translation

Embracing the dual complexity of decision-making and cities is key to delivering research relevant for policy and action, but it exacerbates the efforts required for the important task of translating research [38,40]. Tailored and streamlined messaging, concrete actions, explanations of uncertainty, and plain-language implications for constituents are essential for effective research translation [26,38]. Perhaps overlooked is the variation in what constitutes evidence for researchers, policymakers, and other stakeholders. Politicians, for example, tend to rely on a good story to communicate policy imperatives, rather than use rigorous scientific analyses as a solid evidence base [33,41]. Researchers on the other hand, while struggling with research translation, typically do not explore story-telling communication practices as helpful ways to explain real-world complexities. 

Community and stakeholder engagement is increasingly understood as a critical element of effective consensus-building and policy implementation. Research, in contrast, has often conceived such activities as an exercise of dissemination to be conducted at the end of a research program. Engagement efforts have often been derailed by ineffective consultation from the beginning. This can be especially salient in marginalized communities, resulting in resistance to change, regardless of the evidence presented. In addition, a lack of designated funding and resources in public health research may well have hindered research programs that fully embrace engagement with stakeholders throughout the research process, from conception to dissemination [29].

## 3. A Way Forward

To embrace real-world complexity and ensure the relevance of their work, researchers must integrate a broad variety of collaborators throughout the research process, including those from different academic disciplines, community-based organizations, the private sector, and government. This transition to a comprehensive research framework needs to be supported by the funding landscape. A range of expertise and real-world experience are necessary to inform each step of research, from conception to dissemination. It is important to integrate perspectives from those involved in the decision-making process, as well as those who study these processes and those impacted by policies, into the design and translation of research. The Wales HIA Support Unit is an example of the infrastructure and platforms required for such integration; it provides accessible information, training, resources and guidance for researchers, policy makers, and community members, who may be interested in the HIA process [42].

With growing attention on the climate emergency and opportunities for discussion across sectors, disciplines and regions accorded by COP26 and future COPs, the time is ripe to focus on new ways for research and policy to interact to create greater collective impact. In the last few decades, we have progressively seen a shift towards recognizing health as an important element in policymaking, including climate action. For example, the Health in All Policies (HiAP) movement, which recognizes the complexity of health and demands the systematic integration of health action into all decision-makings across sectors and policy areas, has recently gained momentum [43]. Recently, in an extraordinary move, 200 journals joined up to call for governments to take urgent action on climate change to protect public health [44]. As countries and international bodies plan their strategies for a post-pandemic recovery, plans such as Biden’s Build Back Better plan [45] or the European Green Deal [46], present a prospect for effectively integrating health in their efforts. COP agreements and related activities, along with these recent trends and pledges, provide a great opportunity to take on transformative action for a successful transition towards a more just, healthy and sustainable society. We believe scientists have a role to play in pushing forward a coordinated research, policy and action agenda that leads to the implementation of ambitious programs, plans and strategies to benefit the health of people and the planet. Here, we propose an approach based on co-production and systems thinking. To embrace the interconnected nature of environment and health disciplines and use the existing policy relevant evidence to the greatest advantage, we suggest that the scientific community act to systematically co-produce research on climate policies and engage in systems thinking. 

Co-production provides knowledge and solutions that are developed with different stakeholders and end-users’ inputs: from asking the right questions to identifying relevant outcomes, integrating complex processes of policy making in the research design, and disseminating findings. Co-produced research can ensure the evidence is accessible, useful, and applied in the real world. Collaborating across sectors and disciplines will help break silos, so that policy targets, such as each of the COP26 Campaign Aims, are not treated in isolation but are integrated within a broader goal of promoting health. For example, in the context of COP26, the Clean Transport Aim can promote physical activity and create space for greenspace, which is synergistic with the Nature Aim, which also promotes beneficial exposure to greenspace and cools down cities. 

Within a co-creation framework, using systematic and rigorous methods to gather, process and analyze perspectives from all stakeholders ensures transparency and widespread acceptance. For instance, structured decision-making processes that begin with identifying values, or fundamental objectives of stakeholders can help tease out common goals and trade-offs [47]. It might highlight, for example, how promoting electric vehicles to reduce GHG, while certainly forming part of the solutions and being potentially more politically feasible than radical urban transformations to reduce car use, may have negative impacts in communities where cobalt is mined to produce the vehicle batteries, or create permanent transport infrastructure that is not desirable in the long term for physical or mental health (e.g., maintaining present sedentary commuting patterns), and also still emit harmful non-exhaust emissions [48]. Furthermore, the inclusion of all people potentially affected by a planning decision will ensure greater buy-in and consensus around solutions. For example, the identification of health as an agreed common goal may bring stakeholders traditionally concerned only with narrower outcomes such as transport efficiency or air quality together with experts in health systems and health promotion. Additionally, co-production can help to articulate the type of knowledge or ways to present knowledge that will translate effectively into changes at the local government level, and to encourage public support for transformative policies, programs and/or initiatives. The involvement of a variety of actors can boost inspiration and fundamentally change the vision of cities, while providing healthy criticism and discussions of research assumptions and findings [39].

Systems thinking will ensure the broad and complex variety of pathways to health are considered in the policy decision-making process. It can be defined as “a set of ‘synergistic analytic skills’ used to help describe a complex set of interacting factors that produce outcomes, to predict their behavior and to formulate interventions to achieve desired (and avoid pernicious) results” [30]. Systems thinking can therefore enable transparent and dynamic linkages to be made between politics, ideology, culture, individual and collective behavior, and drivers of health [30]. It ensures drivers and feedback effects are assessed throughout and helps prevent tackling solely symptoms of climate change rather than deeply rooted causes [49]. This will avoid treatments that simply replace one ailment with another, as was the case with diesel engines replacing petrol engines to reduce greenhouse gas emissions. Systems thinking may prevent worse outcomes, such as lock-ins that hinder future actions [37]. For example, investing in electrification of the vehicle fleet in cities can be an “opportunity cost”, i.e. divesting away from far more transformative actions that could help break away from car-reliance in cities. It would also create hard-wired infrastructure that will be part of the urban landscape for years to come. 

Research in public health has always suffered from miniscule financial support, which shrinks even smaller if narrowed to climate change and health funding. Funding opportunities tend to be specific to the interests of the funders, which translate into narrow, siloed projects being supported. Finally, funding rarely supports co-production or holistic approaches. Therefore, it is imperative that public health funding bodies embrace the complexity of public health research and provide the funding to support it [29].

## 4. Conclusions 

Climate change, air pollution and ecologic crises are some of the most concerning public health challenges that we will contend with in the decades to come. Yet, they also present an opportunity to rethink policies and initiatives to embrace health and wellbeing as a primary goal of basic local government functions, such as urban planning, social service delivery, sustainability, education, and economic development. The problems are well understood, and solutions are available. Researchers, policymakers, and stakeholders across sectors and disciplines must start working together to create transformative action that can reshape our cities and living spaces by putting people first. Investing in myopic technocratic solutions misses a great opportunity for health promotion and environmental improvement for all. Climate resilience can be built into urban development plans through policies that enable short-term and long-term health benefits for all people, such as safe active transport networks, public open spaces that offer opportunities for social interaction, accessible public transport, accessible greenspaces, low-carbon healthy food availability, sanitation, and clean air and water, among others. Co-production and systems thinking are holistic approaches that researchers, policymakers and stakeholders alike can embrace to create a common language and form partnerships to build a healthier future for people and the planet. 

## Data Availability

Not applicable.
